# Successful direct acting antiviral (DAA) treatment of HCV/HIV-coinfected patients before and after liver transplantation

**DOI:** 10.1371/journal.pone.0197544

**Published:** 2018-06-06

**Authors:** Julia M. Grottenthaler, Christoph R. Werner, Martina Steurer, Ulrich Spengler, Thomas Berg, Cornelius Engelmann, Heiner Wedemeyer, Thomas von Hahn, Wolfgang Stremmel, Anita Pathil, Ulrich Seybold, Eckart Schott, Usha Blessin, Christoph Sarrazin, Martin-Walter Welker, Ellen Harrer, Stefan Scholten, Clemens Hinterleitner, Ulrich M. Lauer, Nisar P. Malek, Christoph P. Berg

**Affiliations:** 1 Department of Gastroenterology, Hepatology, and Infectiology, University Hospital Tuebingen, Tuebingen, Germany; 2 German Center for Infection Research (DZIF) partner site, Tuebingen, Bonn, Hannover, Heidelberg, Munich, Germany; 3 Department of Internal Medicine I, University of Bonn, Bonn, Germany; 4 Division of Gastroenterology and Hepatology, University Hospital Leipzig, Leipzig, Germany; 5 Department of Gastroenterology, Hepatology, and Endocrinology, Medizinische Hochschule Hannover, Hannover, Germany; 6 Department of Internal Medicine IV, University Hospital Heidelberg, Heidelberg, Germany; 7 Division of Infectious Diseases, Medizinische Poliklinik-Innenstadt, University of Munich, Munich, Germany; 8 Department of Hepatology and Gastroenterology, Charité Universitätsmedizin Berlin, Berlin, Germany; 9 Department of Gastroenterology and Hepatology, University Hospital Frankfurt, Frankfurt, Germany; 10 Department of Internal Medicine 3, Institute of Clinical Immunology, University Hospital Erlangen, Erlangen, Germany; 11 Praxis Hohenstaufenring, Cologne, Germany; 12 Department of Medical Oncology, Haematology, Immunology, Rheumatology and Pulmology, University Hospital Tuebingen, Tuebingen, Germany; 13 Department of Clinical Tumor Biology, University Hospital Tuebingen, Tuebingen, Germany; National Taiwan University Hospital, TAIWAN

## Abstract

**Objectives:**

The aim of this multicenter retrospective study was to investigate safety and efficacy of direct acting antiviral (DAA) treatment in the rare subgroup of patients with HCV/HIV-coinfection and advanced liver cirrhosis on the liver transplant waiting list or after liver transplantation, respectively.

**Methods:**

When contacting 54 German liver centers (including all 23 German liver transplant centers), 12 HCV/HIV-coinfected patients on antiretroviral combination therapy were reported having received additional DAA therapy while being on the waiting list for liver transplantation (patient characteristics: Child-Pugh A (n = 6), B (n = 5), C (n = 1); MELD range 7–21; HCC (n = 2); HCV genotype 1a (n = 8), 1b (n = 2), 4 (n = 2)). Furthermore, 2 HCV/HIV-coinfected patients were denoted having received DAA therapy after liver transplantation (characteristics: HCV genotype 1a (n = 1), 4 (n = 1)).

**Results:**

Applied DAA regimens were SOF/DAC (n = 7), SOF/LDV/RBV (n = 3), SOF/RBV (n = 3), PTV/r/OBV/DSV (n = 1), or PTV/r/OBV/DSV/RBV (n = 1), respectively. All patients achieved SVR 12, in the end. In one patient, HCV relapse occurred after 24 weeks of SOF/DAC therapy; subsequent treatment with 12 weeks PTV/r/OBV/DSV achieved SVR 12. One patient underwent liver transplantation while on DAA treatment. Analysis of liver function revealed either stable parameters or even significant improvement during DAA therapy and in follow-up. MELD scores were found to improve in 9/13 therapies in patients on the waiting list for liver transplantation; in only 2 patients a moderate increase of MELD scores persisted at the end of follow-up.

**Conclusion:**

DAA treatment was safe and highly effective in this nation-wide cohort of patients with HCV/HIV-coinfection awaiting liver transplantation or being transplanted.

## Introduction

Presumably 2.3 million Hepatitis C (HCV) positive patients worldwide are coinfected with human immunodeficiency virus (HIV), of which about 100,000 are living in Western and Central Europe [1]. Coinfection with HIV is known to accelerate complications of chronic HCV infection [2], such as the development and decompensation of liver cirrhosis and emergence of hepatocellular carcinoma (HCC). With the development of direct acting antiviral agents (DAA), antiviral treatment has led to very high SVR (sustained virologic response) rates of 95–100% in the past few years [3–8].

By now, substantial experience exists for DAA therapy in a variety of different patient subgroups. In the special subgroup of HCV/HIV-coinfected patients, DAA treatment shows safety profiles and SVR rates that are nearly identical to those of HCV-monoinfected patients [9–14], although drug-drug interactions (DDIs) with antiretroviral medication can be challenging in this cohort [15, 16]. There have also been a number of trials to evaluate DAA therapy in patients with compensated (Child-Pugh A) liver cirrhosis [17–22], demonstrating favorable safety and efficacy profiles (SVR rates 90–95%) in HCV-monoinfected as well as in HCV/HIV-coinfected patients [11, 12, 23]. Similar SVR rates were demonstrated for HCV-monoinfected patients after liver transplantation [22, 24–31]. In contrast, SVR rates are less impressive in patients with decompensated liver cirrhosis, mainly in the range of 80–85% [3, 4, 17, 22, 26, 27, 32, 33]. Furthermore, the potential of severe deterioration of liver function under DAA therapy in patients with advanced liver cirrhosis [17, 22, 24, 27, 32, 34] has led to the recommendation of very careful usage or even refusal of DAA therapy in patients with MELD (model for end-stage liver disease) scores exceeding 18–20 points [3, 35].

In this context, so far only few data exist concerning safety and efficacy of DAA therapy in HCV/HIV-coinfected patients (i) exhibiting advanced stages of liver cirrhosis while waiting for liver transplantation or (ii) undergoing DAA therapy after liver transplantation. Therefore, we performed a retrospective multicenter analysis throughout Germany, focusing on patients with either of these two rare conditions.

## Patients and methods

For this retrospective cross-sectional multicenter analysis, 54 specialized liver centers in Germany were contacted, including (i) all 23 German university hospitals performing liver transplantations, (ii) 2 additional non-transplanting university hospitals, and (iii) 29 medical practices specialized in hepatology or infectious diseases. Responses were obtained from 22 university hospitals and 10 practices. Notably, 9 university hospitals and 1 medical practice were able to contribute eligible patients to this study. The other institutions reported to have no suitable patients. Data acquisition was carried out pseudonymously by use of a precast Excel chart to ensure structured and standardized data collection. Collected data contained basic patient characteristics, stage of liver disease, specifications of HCV and HIV disease, course of DAA therapy, as well as laboratory results. Recorded time spans encompassed initiation of DAA therapy (baseline) until end of follow-up (12 or 24 weeks after end of treatment).

Complete data sets were obtained for 14 patients. One patient (Pt. 6) received a second DAA therapy, so that a total of 15 DAA therapies were recorded as shown in Table 1. Twelve patients were on the waiting list for liver transplantation at the initiation of DAA therapy with Child-Pugh stage A (n = 6), B (n = 5), C (n = 1); MELD scores: range 7–21, median 13; two patients with HCC; HCV genotypes 1a (n = 8), 1b (n = 2), 4 (n = 2); one patient underwent liver transplantation while on DAA therapy. The decision for listing the individual patients for liver transplantation was in the responsibility of the respective transplant center. Two patients with HCV genotype 1a or 4, respectively, had already been transplanted at the beginning of DAA treatment; one transplanted patient (Pt. 13) suffered from fibrosing cholestatic hepatitis following recurrence of hepatitis C, the other (Pt. 14) from re-cirrhosis of the liver graft. Nine patients had received previous treatment for HCV with Interferon-based therapies. All patients suffered from HIV-1 and were under stable antiretroviral therapy: HIV stadium A1-C3; CD4 cell count 50-1010/μl (CD4 cell count available for n = 11 patients). For more details see Table 1.

**Table 1:** Individual listing of patient characteristics.

Patient	Pt. 1	Pt. 2	Pt. 3	Pt. 4	Pt. 5	Pt. 6a	Pt. 6b^a^	Pt. 7	Pt. 8	Pt. 9	Pt. 10	Pt. 11	Pt. 12	Pt. 13	Pt. 14
LTx-Status	pre	pre	pre	pre	pre	pre	pre	pre	pre	pre	pre	pre	pre	post	post
	LTx	LTx	LTx	LTx	LTx	LTx	LTx	LTx	LTx	LTx	LTx	LTx	LTx	LTx	LTx^b^
**General data**															
Age (years)	45	57	49	56	48	64	64	39	49	53	48	54	38	45	49
Sex	m	f	f	m	m	m	m	m	m	m	m	m	m	m	m
BMI (kg/m^2^)	33	19	23	26	25	25	25	21	23	35	21	17	21	22	23
Child-Pugh	C	A	B	B	B	B	B	A	A	A	A	A	B	n/a	un-known
MELD	18	9	9	10	13	16	14	21	13	12	7	14	17	12	13
HCC	no	yes	no	no	no	no	no	no	no	no	no	no	yes	no	no
Time since LTx	n/a	n/a	n/a	n/a	n/a	n/a	n/a	n/a	n/a	n/a	n/a	n/a	0^c^	35	58
(months)															
Immuno-suppressants	n/a	n/a	n/a	n/a	n/a	n/a	n/a	n/a	n/a	n/a	n/a	n/a	TAC MMF Pred	CSA MMF Pred	TAC MMF -
**HCV**															
Genotype	4	1a	1a	1b	4	1b	1b	1a	1a	1a	1a	1a	1a	4	1a
First diagnosed	8	31	18	32	30	18	18	35	21	21	un-known	21	~30	9	11
(… years ago)															
Pretreatment with IFN-based regimen	no	no	no	yes	no	yes	yes	yes	yes	yes	no	yes	yes	yes	yes
or DAA therapy	no	no	no	no	no	no	yes	no	no	no	no	no	no	no	no
Outcome of previous treatment	n/a	n/a	n/a	NR	n/a	discontinuation	relapse after DAA therapy	relapse	relapse	NR	n/a	NR	NR	NR	NR
**HIV**															
HIV type	HIV-1	HIV-1	HIV-1	HIV-1	HIV-1	HIV-1	HIV-1	HIV-1	HIV-1	HIV-1	HIV-1	HIV-1	HIV-1	HIV-1	HIV-1
First diagnosed	18	31	30	32	30	17	17	35	26	27	26	27	~30	11	15
(… years ago)															
Stadium	A2	A1	B3	C2	B3	B2	B2	C3	B3	A1	C2	C3	B3	A1	A2
HIV-Medication	FTC/TDF/RPV	FTC/TDF/ATV/RTV	3TC/FPV/RTV	FTC/TDF/LPV/RTV	ATV	FTC/TDF/FPV/RTV	FTC/TDF/FPV/RTV	DRV/RTV	FTC/TDF/RAL	FTC/TDF/RAL	DRV/RAL/RTV	LPV/RTV/3TC/ABC/RAL	FTC/TDF/RAL	3TC/ABC/RAL	TDF/3TC/RAL
**DAA therapy**															
HCV-Medication	SOF/DAC	SOF/DAC	SOF/LDV/RBV	SOF/DAC	SOF/DAC	SOF/DAC	PTV/r/ OBV/ DSV	PTV/r/ OBV/DSV/RBV	SOF/RBV	SOF/DAC	SOF/LDV/RBV	SOF/LDV/RBV	SOF/RBV	SOF/DAC	SOF/RBV
Duration (weeks)	24	24	12	12	24	24	12	24	24	24	24	24	27	40^d^	24
HCV-RNA-PCR first negative (weeks)	4	8	2	12	8	4	4	8	12	12	24	24	6	27	8
Outcome	SVR 12	SVR 12	SVR 12	SVR 12	SVR 12	relapse	SVR 12	SVR 12	SVR 12	SVR 12	SVR 12	SVR 12	SVR 12	SVR 12	SVR 12
Complications	yes^e^	no	no	no	no	no	no	no	no	no	no	yes^f^	yes^g^	yes^h^	yes^i^
Transplant rejection during DAA therapy	n/a	n/a	n/a	n/a	n/a	n/a	n/a	n/a	n/a	n/a	n/a	n/a	no	no	no

Pt. 1–14 = Patients 1–14; LTx = liver transplantation; pre LTx = patient on liver transplant waiting list at initiation of DAA therapy; post LTx = DAA therapy was initiated after liver transplantation; m = male; f = female; BMI = body mass index; n/a = not applicable; MELD = model for end-stage liver disease; HCC = hepatocellular carcinoma; TAC = Tacrolimus; MMF = Mycophenolate mofetil; Pred = Prednisolone; CSA = Ciclosporin; IFN = Interferon; NR = nonresponse; FTC = Emtricitabine; TDF = Tenofovir; RPV = Rilpivirine; ATV = Atazanavir; RTV = Ritonavir; 3TC = Lamivudine; FPV = Fosamprenavir; LPV = Lopinavir; DRV = Darunavir; RAL = Raltegravir; ABC = Abacavir; SOF = Sofosbuvir; DAC = Daclatasvir; LDV = Ledipasvir; RBV = Ribavirin; PTV = Paritaprevir; r = Ritonavir; OBV = Ombitasvir; DSV = Dasabuvir; PCR = polymerase chain reaction; SVR 12 = sustained virologic response 12 weeks after end of treatment.

^a^ relapse after SOF/DAC (24 weeks), SVR 12 achieved after second therapy with PTV/r/OBV/DSV (12 weeks);

^b^ re-cirrhosis in transplanted liver;

^c^ LTx three weeks after initiation of DAA therapy, initial immunosuppressive therapy with TAC/MMF/Pred, TAC switched to Sirolimus during DAA therapy;

^d^ 40 weeks of therapy, addition of RBV after three months (discontinuation after 14 days due to worsening of renal function), and SMV after four months, respectively, due to a delayed drop in HCV RNA levels;

^e^ biliary stent occlusion with cholangitis;

^f^ hemolytic anemia, resulting in discontinuation of RBV;

^g^ HCC recurrence after LTx;

^h^ fibrosing cholestatic hepatitis, renal insufficiency, incisional hernia;

^i^ abdominal pain.

Data analysis was performed using IBM SPSS Statistics (Version 24), GraphPad Prism (Version 7.03), and Microsoft Excel (Version 2010). Continuous variables in dependent samples were analyzed with Friedman’s ANOVA. P values < 0.05 were considered significant. For post hoc analysis, Wilcoxon signed ranks test was used. Bonferroni-Holm correction was applied in case of multiple comparisons. Retrospective simulation of potential DDIs was performed by use of the website www.hep-druginteractions.org (University of Liverpool) [3]. HCV RNA negativity was defined as HCV RNA PCR below the lower limit of detection in a sensitive assay.

This study was approved by the Ethics Committee at the Medical Faculty of the Eberhard-Karls University and at the University Hospital Tuebingen (Project number: 805/2015BO2). The Committee waived the need for written informed consent for this retrospective analysis.

## Results

DAA treatment regimens were chosen as shown in Table 1. Regimen choices were based on the available options at the time of initiation of therapy. No patient died during treatment and there was no case, in which DAA treatment had to be discontinued (two discontinuations of Ribavirin). In five patients, complications were recorded as listed in Table 1. One patient underwent liver transplantation while on DAA therapy. There were no transplant rejections during therapy and follow-up. The antiretroviral treatment regimen was modified in one patient (Pt. 13) prior to DAA therapy. Retrospective simulation yielded potential DDIs of DAAs with antiretroviral medication in 11/15 therapy regimens (73.3%). Serum levels of antiretroviral medication were monitored in one patient. HIV RNA PCR was performed in 12/14 patients with varying frequency at non-standardized time-points. No significant HIV viremia or viral blips were detected during DAA therapy. In the follow-up phase, only one transient episode of low level HIV viremia was reported (Pt. 1, 89 copies/ml).

**Fig 1:**
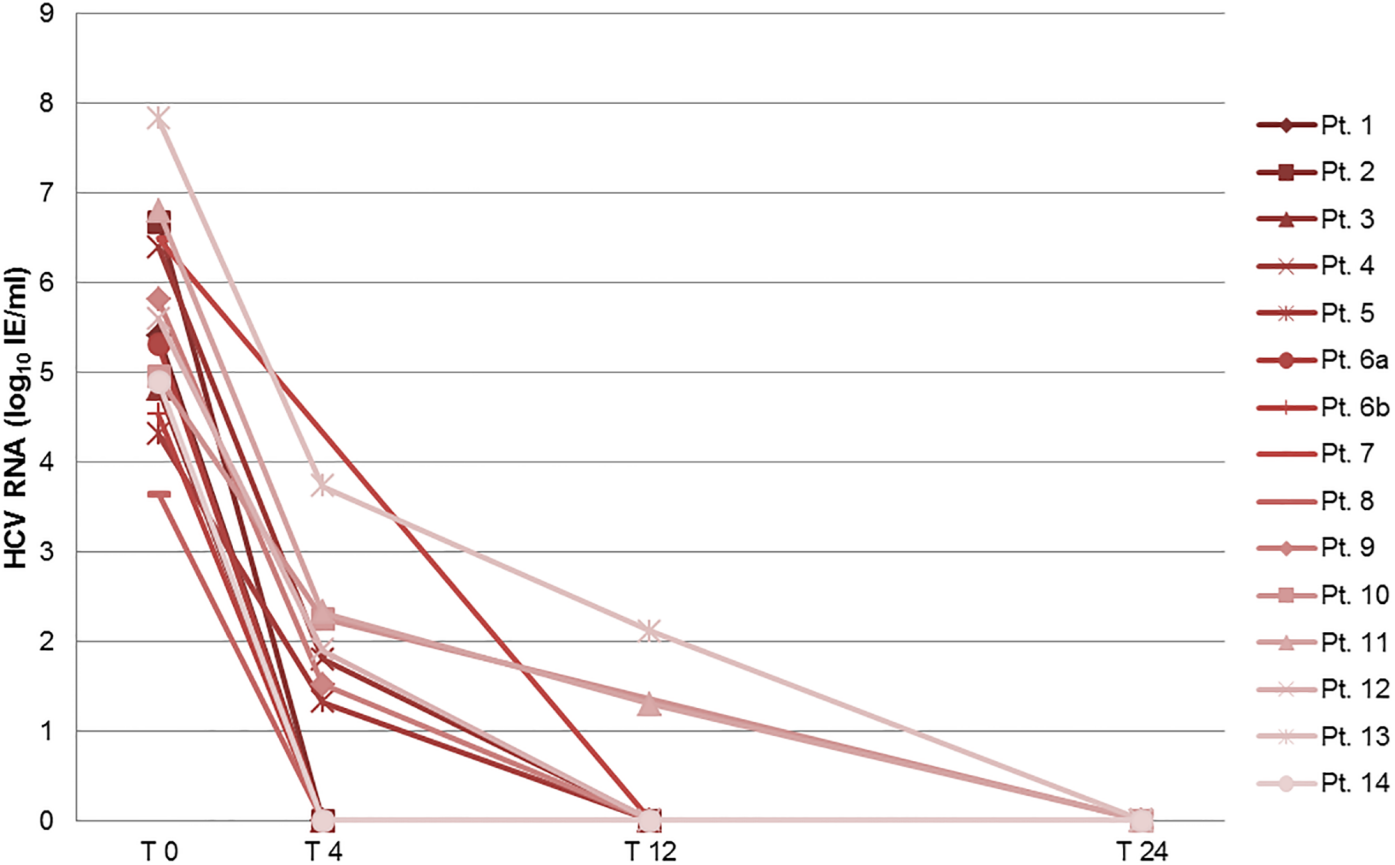
Course of HCV RNA under DAA therapy. Pt. 1–14: individual patients. T 0 = initiation of DAA therapy; T 4/12/24 = 4/12/24 weeks after initiation of DAA treatment (T24 only if treatment course was long enough). Undetectable HCV RNA was set to zero in the otherwise logarithmized data.

Notably, 13/14 patients achieved SVR 12 following the first DAA therapy (92.9%; 11 patients on the transplant waiting list and both patients after liver transplantation). HCV RNA negativity was reached after a median of 8 weeks during DAA therapy (range 2–27 weeks; see Fig 1). One patient (No. 6a) suffered from a relapse following 24 weeks of therapy with Sofosbuvir / Daclatasvir; subsequently, this patient achieved SVR 12 under a modified DAA therapy with Paritaprevir / Ritonavir / Ombitasvir / Dasabuvir, administered over 12 weeks (see Pt. 6b).

**Fig 2:**
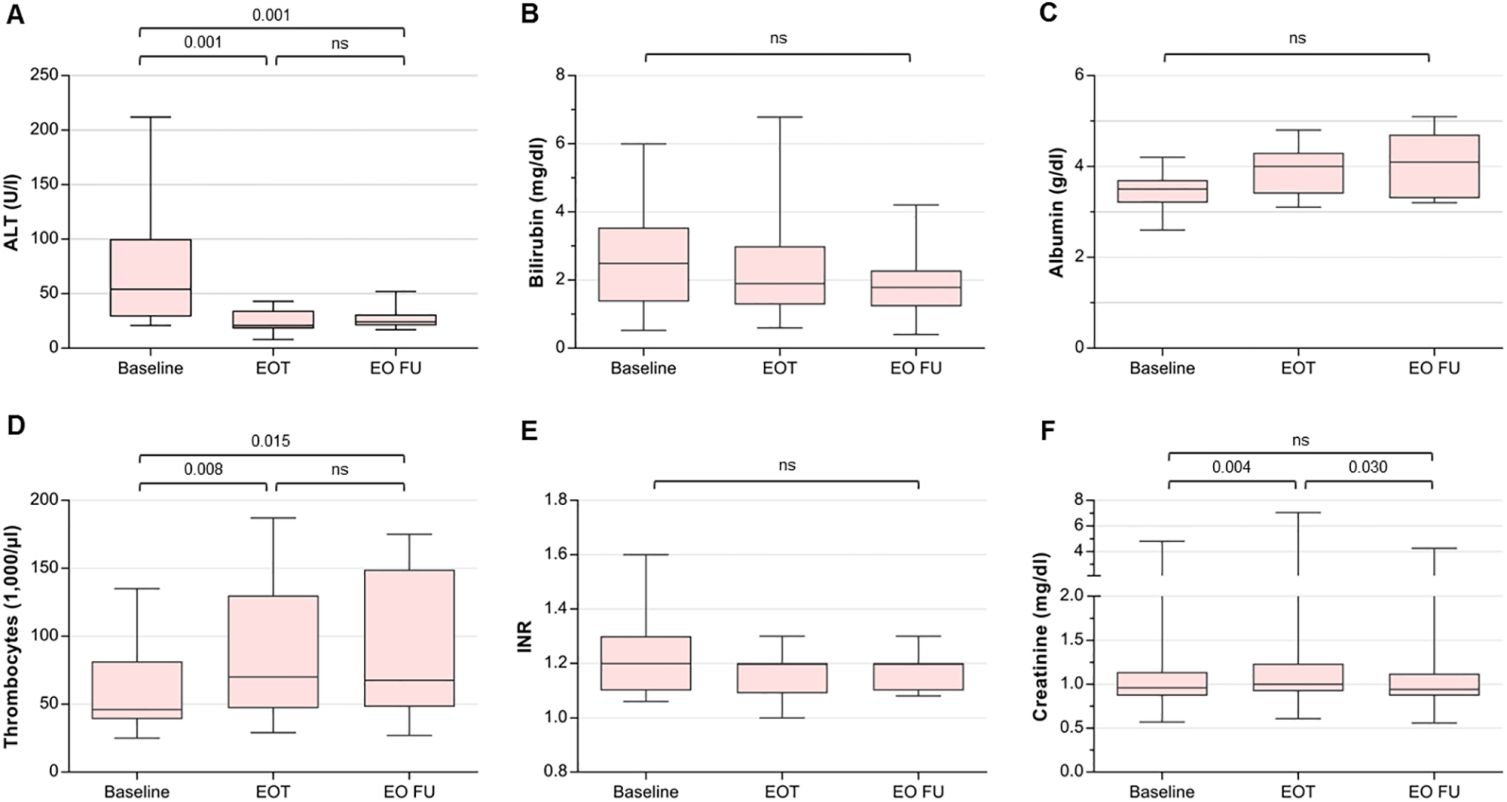
Changes of (A) alanine transaminase (ALT), (B) bilirubin, (C) albumin, (D) thrombocytes, (E) INR (international normalized ratio), and (F) creatinine in patients on the liver transplant waiting list. Box plots: median and interquartile ranges; whiskers: minimum to maximum. P-values from post hoc analysis (Wilcoxon signed ranks test, Bonferroni-Holm correction); ns = not significant, EOT = end of treatment, EO FU = end of follow-up.

For safety analysis, we monitored the course of liver function tests and MELD scores under DAA therapy and during follow-up. Statistical analysis was performed on the results of the patients on the transplant waiting list (see Table 2 and Fig 2). In all evaluable cases, significant and persistent improvements of serum alanine transaminase (p < 0.001) and thrombocyte levels (p = 0.002) could be observed. INR (international normalized ratio), bilirubin, and albumin displayed a trend towards improvement without reaching levels of significance. Creatinine levels (p = 0.002) and eGFR (estimated glomerular filtration rate; p = 0.013) showed minimal changes of the mean, however, we could demonstrate a transient, statistically significant worsening under DAA therapy. These changes remained significant even when excluding the one patient (Pt. 7) with a markedly impaired and fluctuating renal function from analysis on a trial basis. The two transplanted patients showed stable liver function tests under DAA therapy and during follow-up. Especially the patient suffering from fibrosing cholestatic hepatitis (Pt. 13) displayed a pronounced improvement in bilirubin levels, albumin, and thrombocytes.

**Table 2:** Changes in laboratory results under DAA therapy and during follow-up in patients on the liver transplant waiting list.

Parameter		N	Baseline	EOT	EO FU	p-value
**ALT**	Mean ± SEM	13	70 ± 14.7	24 ± 2.9	27 ± 2.6	**<0.001**
(U/l)	Range		21–212	8–43	17–52	
**Thrombocytes**	Mean ± SEM	12	60 ± 10.0	84 ± 14.0	92 ± 15.5	**0.002**
**(**1,000/μl)	Range		25–135	29–187	27–175	
**MELD**	Mean ± SEM	11	14 ± 1.2	13 ± 1.7	11 ± 1.4	0.307
	Range		7–21	6–27	5–22	
**Bilirubin (total)**	Mean ± SEM	13	2.6 ± 0.4	2.5 ± 0.5	1.9 ± 0.3	0.101
(mg/dl)	Range		0.5–6.0	0.6–6.8	0.4–4.2	
**INR**	Mean ± SEM	13	1.2 ± 0.05	1.2 ± 0.02	1.2 ± 0.02	0.832
	Range		1.1–1.6	1.0–1.3	1.1–1.3	
**Albumin**	Mean ± SEM	9	3.4 ± 0.2	3.9 ± 0.2	4.0 ± 0.2	0.169
(g/dl)	Range		2.6–4.2	3.2–4.8	2.7–5.1	
**Creatinine**	Mean ± SEM	13	1.3 ± 0.3	1.5 ± 0.5	1.2 ± 0.3	**0.002**
(mg/dl)	Range		0.6–4.8	0.6–7.1	0.6–4.3	
**eGFR**	Mean ± SEM	13	84 ± 27.6	79 ± 27.9	86 ± 25.7	**0.013**
(ml/min/1,73m^2^)	Range		14–119	9–116	16–119	

Analysis was only possible for data sets, in which values for all three time points (baseline, end of treatment (EOT), end of follow-up (EO FU)) were given. Significant p-values (Friedman’s ANOVA) are printed in bold. Mean, standard error of the mean (SEM), and range are indicated for the three time points. N = number of data sets of DAA therapies which could be analyzed per parameter; ALT = alanine transaminase; MELD = model for end-stage liver disease; INR = international normalized ratio; eGFR = estimated glomerular filtration rate. Calculation of eGFR by use of the Chronic Kidney Disease-Epidemiology Collaboration (CKD-EPI) equation [36].

**Fig 3:**
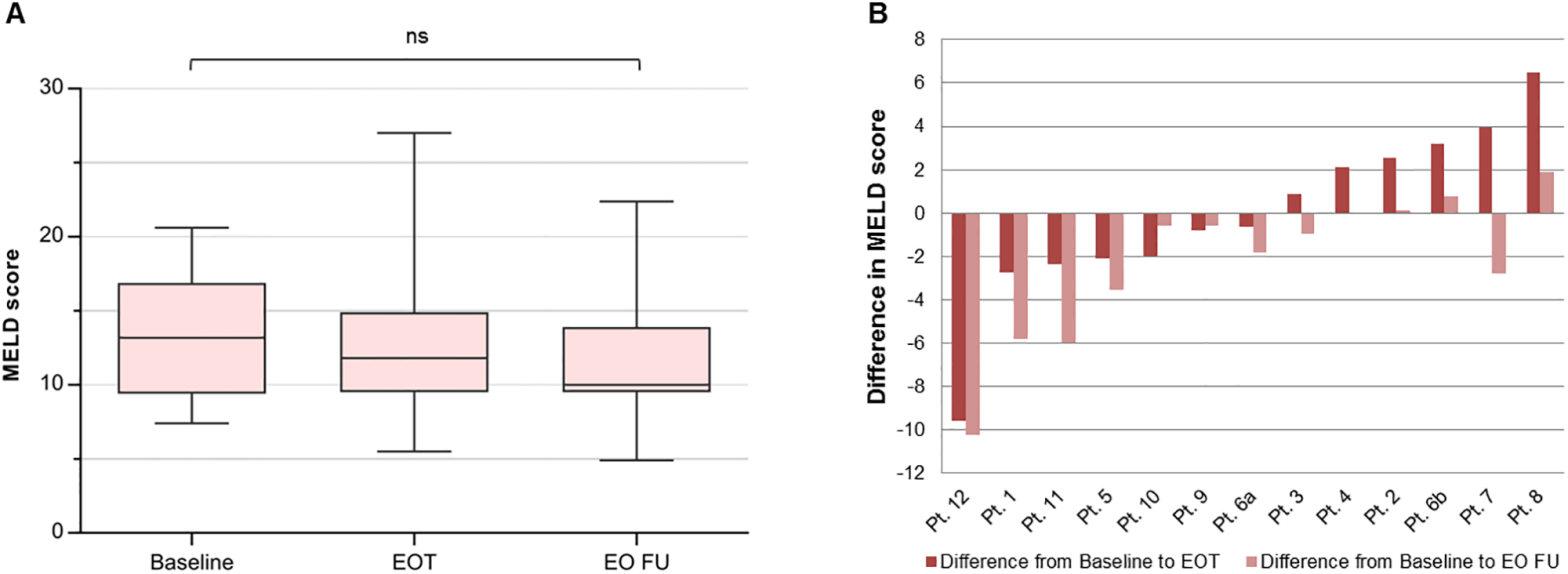
**(A) Changes of MELD scores in patients on the liver transplant waiting list.** Changes did not reach significance levels; ns = not significant, EOT = end of treatment, EO FU = end of follow-up. **(B) Changes of individual MELD scores in patients on the liver transplant waiting list**. Pt. 1–12: individual patients. EOT = end of treatment, EO FU = end of follow-up.

As shown in Fig 3A, initial individual MELD scores improved in 9 patients on the transplant waiting list compared to baseline: 7 patients showed sustained improvement from the DAA treatment period until the end of follow-up, while 2 patients started to improve only when reaching the follow-up period. One of these patients’ (Pt. 7) MELD score was mainly driven by an impaired and fluctuating renal function. In contrast, 2 patients showed worsening of their MELD scores persisting throughout treatment and follow-up. MELD scores in another 2 patients returned to baseline at the follow-up period after initial worsening under DAA therapy. Overall, changes in MELD scores did not reach a level of significance (see Table 2 and Fig 3B).

## Discussion

In this retrospective German multicenter study, data on safety and efficacy of DAA therapy in patients with HCV/HIV-coinfection on the liver transplant waiting list or after liver transplantation, respectively, were analyzed. SVR rates in this rare patient subgroup were encouragingly high: Although a substantial number of patients with decompensated liver cirrhosis (50% Child-Pugh B/C) were included, all chronic HCV infections were cured, including the one patient who achieved SVR only after a second, modified DAA treatment. Our results seem to slightly exceed SVR rates in the one and only case series including HCV/HIV-coinfected patients on the liver transplant waiting list, which has yet been published [37]. This study reported an overall SVR rate of 85.4% (79.2% pre liver transplantation) while not discussing the possibility for re-treatment in case of failure. Our data are also slightly exceeding results for DAA treatment in advanced liver disease for HCV-monoinfected patients in prospective trials such as SOLAR-1 [26], SOLAR-2 [27], ASTRAL-4 [32], CUP [17], ALLY-1 [22], as well as in larger real-world cohort analyses [4, 17]. This may be due to the small sample size and highly selected patients in our study. Furthermore, DAA treatment was solely performed in highly specialized and experienced liver centers, which might have contributed to these optimal outcomes.

In addition to the remarkable SVR rates, our data demonstrate a tolerable safety profile of DAA therapy in this special cohort. Most recorded complications show no clear association to antiviral therapy. Moreover, we could demonstrate an overall stability of liver function during DAA therapy and in follow-up. Statistical analysis revealed transient significant worsening of creatinine levels and eGFR under DAA therapy. However, changes of the mean were very small and most patients had normal or nearly normal renal function throughout observation. This caused a pronounced floor effect impairing the impact of this result. Furthermore, in all patients receiving antiretroviral regimens containing Tenofovir, eGFR did not fall below the threshold associated with a risk of accumulation. MELD score changes with approximately half of the patients showing improvement are consistent with data for HCV-monoinfected patients with decompensated liver cirrhosis [32]. Furthermore, the significant and sustained improvement of thrombocyte levels could be taken as an indicator for reduction in portal hypertension. Our findings are in line with data on long-term outcome in HCV-monoinfected patients on the liver transplant waiting list, in which successful DAA therapy can lead to significant improvement and stabilization of liver function, even enabling patients to be removed from the transplant waiting list [33, 35]. Interestingly, there were no reports on any clinically relevant adverse drug effects, although retrospective screening for DDIs of DAAs with the antiretroviral medication yielded potential interactions in 73% of the patients.

There are three reports in the literature [38–40] dealing with the subject of DAA therapy in altogether 15 HCV/HIV-coinfected patients after liver transplantation. High SVR rates, but also several complications, especially one rejection and one case of death [38], were reported. Our two liver transplant patients undergoing DAA therapy both achieved SVR 12 and did not suffer from any severe complication.

Considering the high response rate of the specialized centers, the relatively small number of patients that were detected in our study probably comes close to the actual size of this very rare subgroup of patients in Germany. Our data correspond to different cohort studies stating a prevalence of 2.6–3.0% [41, 42] for HCV/HIV-coinfected patients on the liver transplant waiting list, and a prevalence of 0.2–0.3% [43, 44] for HCV/HIV-coinfected patients after liver transplantation.

Many questions remain to be answered for this special patient collective: Besides providing further proof for safety and efficacy of DAA treatment, future studies should focus on the choice of DAA regimens, the clinical relevance of potential DDIs, as well as the patients’ long-term outcome. Given the low prevalence of HCV/HIV-coinfected patients on the liver transplant waiting list and after liver transplantation, respectively, international studies are required to recruit a sufficient number of patients.

In summary, our data strongly suggest that DAA treatment can be performed with excellent efficacy and tolerable safety in patients with HCV/HIV-coinfection and advanced liver cirrhosis on the liver transplant waiting list as well as in HCV/HIV-coinfected patients after liver transplantation. However, considering this challenging patient population, DAA therapy should preferably be performed in specialized liver centers comprising particular knowledge in managing patients with HIV-infection.
